# An Atypical Presentation of Takayasu Arteritis: Isolated Tinnitus Followed by Ectopic Pregnancy

**DOI:** 10.7759/cureus.91746

**Published:** 2025-09-06

**Authors:** Maria Alsarayreh, Eyad Elfar, Rahaf Khames

**Affiliations:** 1 Dermatology, Private Practice, Orlando, USA; 2 Family Medicine, WinMed Health, Orlando, USA; 3 Medicine, Alfaisal University College of Medicine, Riyadh, SAU

**Keywords:** autoimmune vasculitis, ectopic, hearing loss, pregnancy, pregnancy complications, takayasu arteritis, tinnitus

## Abstract

Takayasu arteritis (TA) is a chronic large-vessel vasculitis that may present with a broad spectrum of clinical manifestations, some of which are subtle or atypical. Otologic symptoms are not commonly emphasized in this context, yet in some cases, they may precede more overt systemic findings, as illustrated in this report. We report a case of a 41-year-old female whose initial symptom was bilateral pulsatile tinnitus, occurring in the absence of hearing loss or audiometric abnormalities. In this patient, the tinnitus persisted for approximately five or six months before the onset of systemic complaints. Serial imaging over time revealed progressive, multi-territorial vascular involvement consistent with TA. Her treatment course included long-term corticosteroid therapy, which was complicated by an ectopic pregnancy requiring coordinated interdisciplinary care. This case highlights the potential relevance of isolated otologic symptoms as an early clinical clue in systemic vasculitis. Such atypical presentations can make the diagnosis more challenging and contribute to delays in treatment. It also underscores the importance of comprehensive management, including reproductive counseling for women of childbearing age and ongoing surveillance.

## Introduction

Takayasu arteritis (TA) is a rare, chronic granulomatous inflammatory condition that primarily involves large arteries, especially the aorta and its main branches. TA is classified as an autoimmune disease, where immune-driven inflammation can lead to significant complications, including arterial stenosis or occlusion, organ damage, and an elevated risk of cardiovascular and cerebrovascular ischemic events [1]. Approximately 80-90% of TA cases occur in women and usually develop between the ages of 10 and 40 years [2]. It presents with a broad spectrum of systemic symptoms, including constitutional signs such as weight loss, low-grade fever, fatigue, and arthralgia. Vascular involvement can result in intermittent claudication, diminished or absent peripheral pulses, and hypertension. Neurological features may include vertigo, syncope, visual disturbances, headache, and stroke [3].

Extracranial auditory manifestations in Takayasu arteritis (TA) are exceedingly rare. A 2021 case report described bilateral sensorineural hearing loss with associated tinnitus as the initial presentation of TA [4], while another detailed recovery from recurrent episodes of sudden hearing loss with persistent low-pitched tinnitus following combined corticosteroid and hyperbaric oxygen therapy [5]. Notably, both cases involved objective auditory deficits. To date, isolated tinnitus without concurrent hearing loss as the sole presenting symptom has not been previously reported, highlighting the atypical nature and diagnostic complexity of the case presented herein.

Notably, the patient developed a left-sided ectopic pregnancy, raising questions about tubal function and reproductive risk in the context of chronic vasculitis and corticosteroid exposure. Here, we describe a 41-year-old woman with a novel dual presentation - initial isolated bilateral tinnitus and a subsequent ectopic pregnancy, both occurring in the setting of confirmed Takayasu arteritis.

## Case presentation

A 41-year-old woman presented in 2025 for hypertension management. She retrospectively described persistent bilateral pulsatile tinnitus, characterized as a "whooshing" sound, most prominent at night and in quiet environments, which had been present since 2017. There was no associated hearing loss, vertigo, imbalance, otalgia, or ear fullness. Five or six months later, systemic symptoms emerged, including fatigue, low-grade fever, arthralgia, headaches, and exertional discomfort in the arms and legs.

Diagnostic studies between 2017 and 2018 revealed unremarkable audiometry, tympanometry, MRI of the internal auditory canal and inner ear, electrocardiogram, and echocardiography. Thoracic computed tomography (CT) showed subtle narrowing of the distal thoracic and abdominal aorta, raising concern for vascular involvement. Positron emission tomography (PET-CT) was recommended but deferred. Carotid duplex ultrasonography demonstrated elevated peak systolic velocities (Table 1).

**Table 1:** Carotid duplex ultrasonography measurements. Peak systolic and end-diastolic velocities for selected carotid vessels are shown. Findings are consistent with significant stenosis. The study was performed at an external facility, and representative Doppler images were not retained at the time of acquisition. CCA: common carotid artery; ICA: internal carotid artery

Vessels	Peak systolic velocity (cm/s)	End-diastolic velocity (cm/s)	Interpretation
Right CCA	347.64	19.36	Significantly elevated - suggests stenosis
Left CCA	354.14	40.34	Elevated - suggestive of narrowing
Right ICA	64.65	15.87	Within normal range
Left ICA	232.76	25.53	Consistent with >70% stenosis

In July 2019, an evaluation conducted in Morocco revealed active large-vessel vasculitis. Physical examination showed arm blood pressure asymmetry (>20 mmHg), a weak right brachial pulse, absent left brachial and pedal pulses, and vascular bruits over the left carotid and subclavian arteries. Inflammatory markers were elevated (ESR and CRP), while complete blood count, renal, and hepatic panels were within normal limits. Autoimmune and infectious screens were negative (Table 2).

**Table 2:** Comprehensive laboratory evaluation. Note: Only selected complete blood count (CBC) parameters are displayed; remaining values were within normal limits and not clinically contributory. ANA: antinuclear antibody; Anti-CCP: anti–cyclic citrullinated peptide; RF: rheumatoid factor; RNP: ribonucleoprotein; Scl-70: scleroderma-70; c-ANCA: cytoplasmic anti-neutrophil cytoplasmic antibodies; p-ANCA: perinuclear anti-neutrophil cytoplasmic antibodies; S: serum; P: plasma; HBsAg: hepatitis B surface antigen; HCV Ab: hepatitis C antibody; VDRL: venereal disease research laboratory; TPHA: treponema pallidum hemagglutination assay; APTT: activated partial thromboplastin time; PT: prothrombin time

Categories	Tests	Results	Reference ranges	Interpretation
Complete blood count	WBC	8.16 ×10³/mm³	4.0-10.0 ×10³/mm³	Normal
	Hemoglobin	13.2 g/dL	12.0-16.0 g/dL	Normal
	Platelets	336 ×10³/mm³	150-450 ×10³/mm³	Normal
Inflammatory markers	ESR	57 mm/h	<20 mm/h	Elevated
	CRP	14 mg/L	≤8.0 mg/L	Elevated
	Ferritin	14 µg/L	11-307 µg/L	Normal
Renal function	Creatinine	0.71 mg/dL	0.60-1.20 mg/dL	Normal
	BUN	17 mg/dL	7-25 mg/dL	Normal
Liver enzymes	Aspartate transaminase (AST)	15 U/L	8-43 U/L	Normal
	Alanine transaminase (ALT)	13 U/L	7-45 U/L	Normal
Autoimmune panel	ANA	0.6 U	≤1.0 U	Negative
	Anti-CCP	<15.6 U	<20 U	Negative
	Rheumatoid factor (RF)	<15 IU/mL	<15 IU/mL	Negative
	SSA/SSB antibodies	Negative	Negative	Negative
	Anti-Smith/RNP/Scl-70/dsDNA	Negative	Negative	All negative
	c-ANCA pattern	Negative	Negative	Negative
	p-ANCA pattern	Negative	Negative	Negative
Antiphospholipid panel	Cardiolipin IgG	<9.4 GPL	<15.0 GPL	Negative
	Cardiolipin IgM	9.6 MPL	<15.0 MPL	Negative
	Lupus anticoagulant	Not detected	Negative	Negative
Cryoproteins	Cryoglobulin, S	Negative	Negative	Negative
	Cryofibrinogen, P	Negative	Negative	Negative
Infectious screen	HIV (Ab/Ag)	0.12	<0.9	Negative
	Hepatitis B surface Ag (HBsAg)	0.00	<0.9	Negative
	Hepatitis C Ab	0.15	<1.0	Negative
	Syphilis (VDRL/TPHA)	Negative	Negative	Negative
Coagulation profile	APTT coagulation study	32 s	25-37 s	Normal
	PT coagulation study	11.2 s	9.4-12.5 s	Normal
	INR	1.00	0.9-1.1	Normal

**Figure 1:**
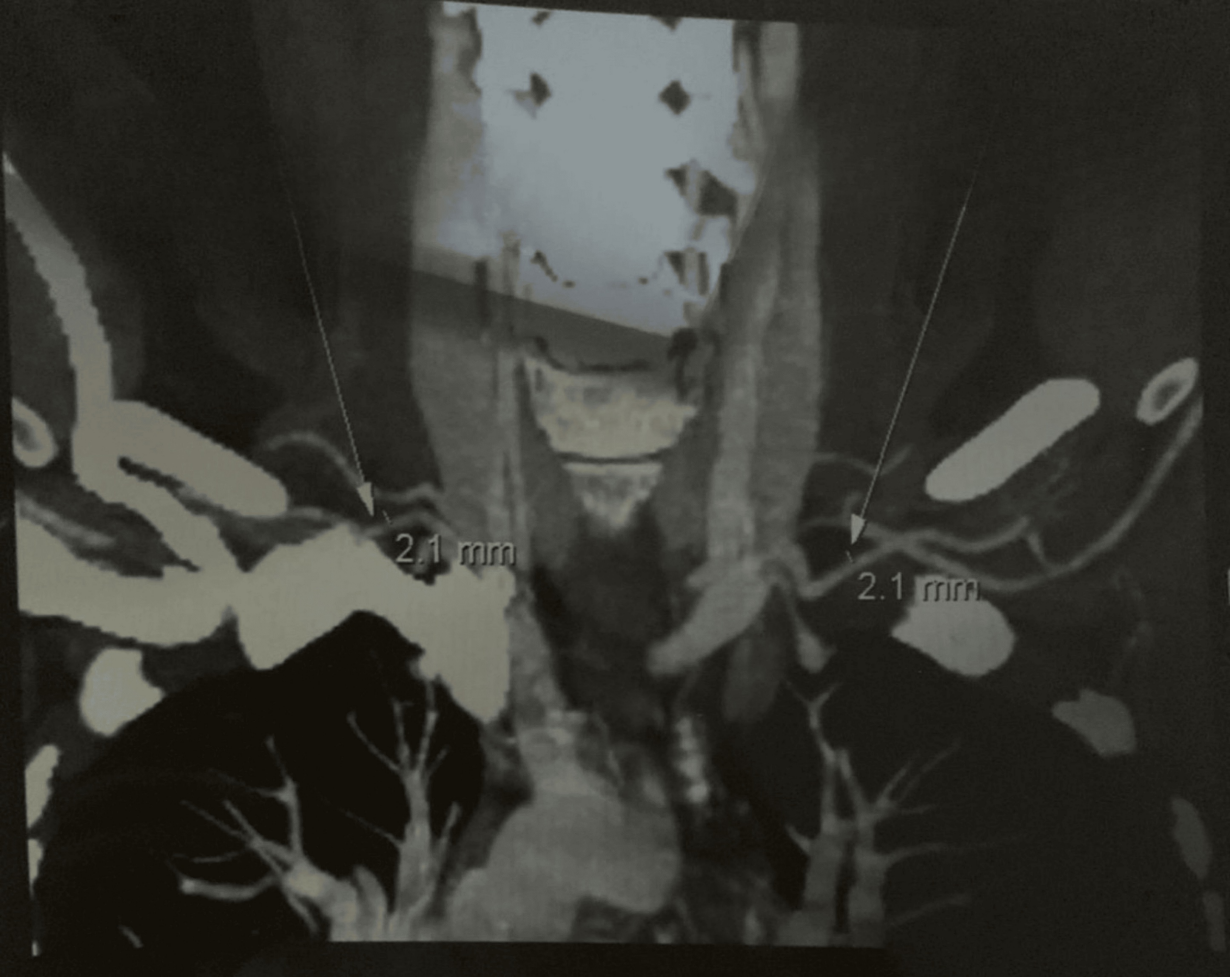
Bilateral subclavian arteries: symmetrical wall thickening. Angiographic imaging of the subclavian arteries demonstrates symmetrical wall thickness of 2.1 mm bilaterally. Arrows indicate the measurement sites.

**Figure 2:**
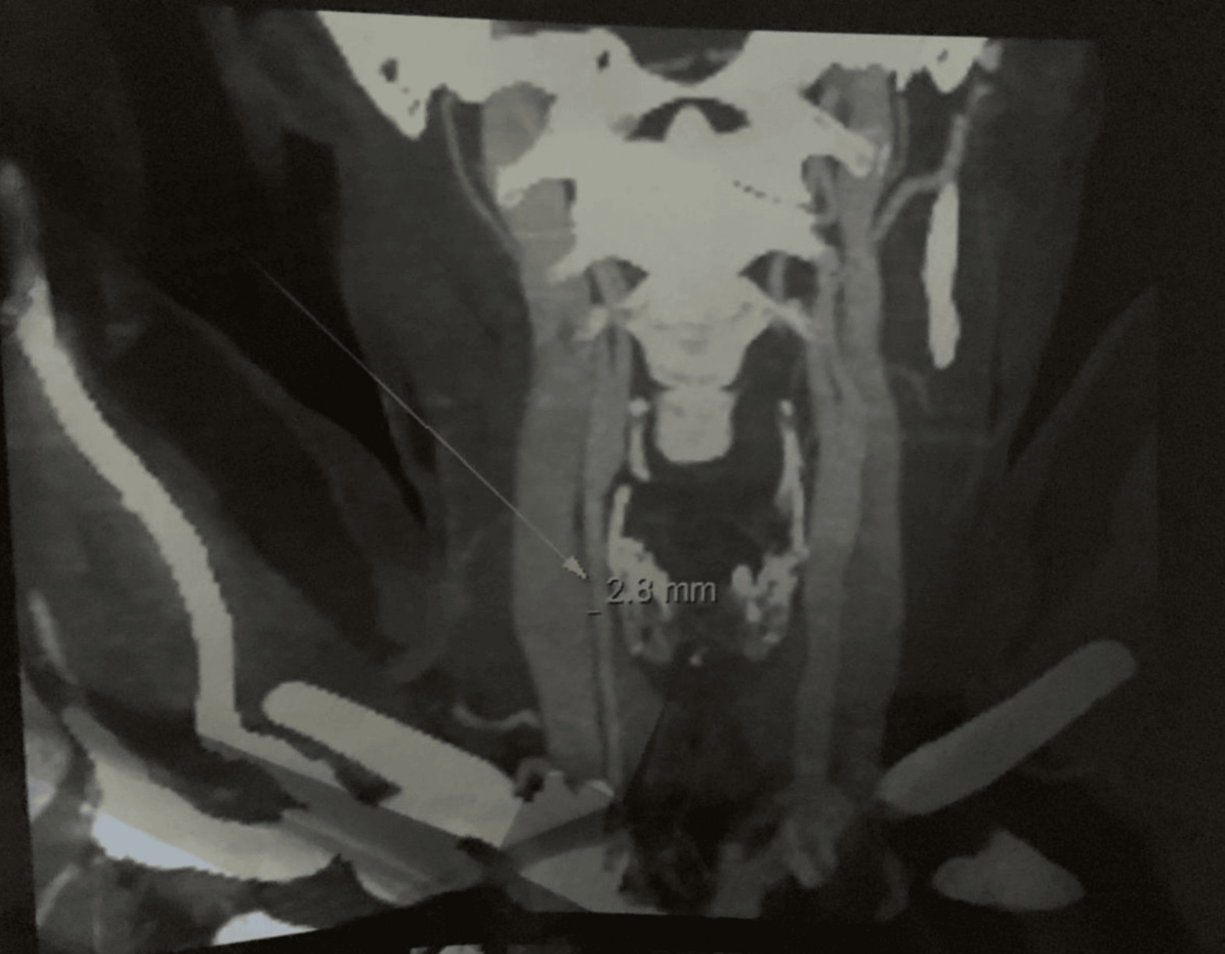
Left common carotid artery: focal wall thickening. Axial CT angiography of the left common carotid artery demonstrates focal mural thickening with a measured wall thickness of 2.8 mm. The arrow indicates the site of measurement along the arterial wall.

**Figure 3:**
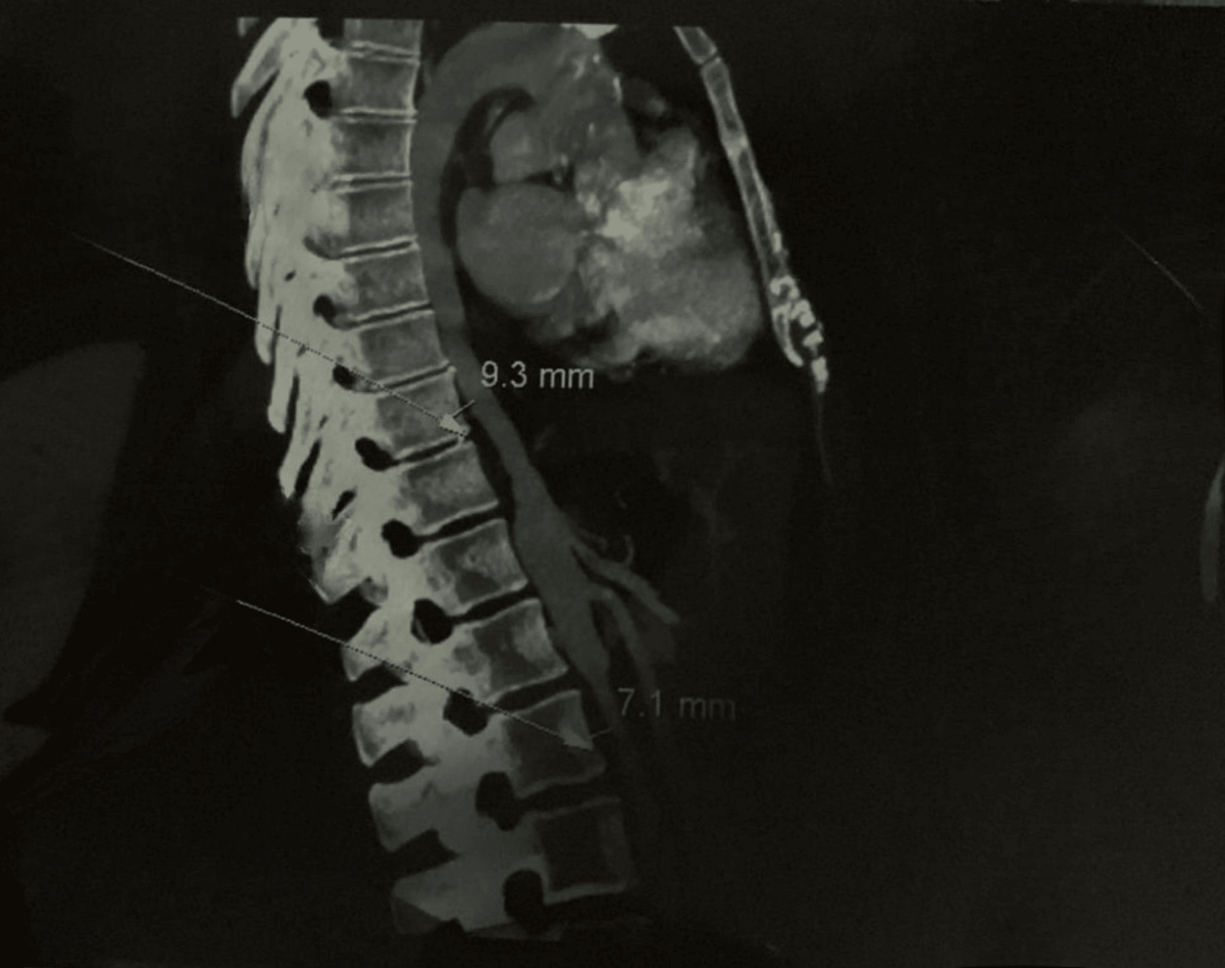
Thoracoabdominal aorta: segmental wall thickening. Axial CT angiography reveals mural thickening of the thoracoabdominal aorta, measuring 9.3 mm in the descending thoracic segment and 7.1 mm in the infrarenal abdominal segment. Arrows indicate the respective sites of measurement.

Contrast-enhanced CT and PET-CT demonstrated mural thickening and luminal narrowing of the thoracic and abdominal aorta, bilateral subclavian arteries, and the left common carotid artery. The principal imaging findings are summarized below each corresponding figure (Figures 1-3). Optical coherence tomography revealed normal retinal structure bilaterally, excluding ocular involvement.

The patient was treated with oral prednisolone (60 mg daily, tapered to 5 mg), plus aspirin and bisoprolol. Systemic symptoms and blood pressure improved, while tinnitus persisted with mild subjective improvement, though it did not fully resolve. Follow-up imaging in 2022 in the United States was compared to 2019 studies performed in Morocco, revealing interval progression across multiple vessels (Table 3).

**Table 3:** Comparative angiographic findings from 2019 to 2022.

Vessel	2019 Findings	2022 Findings	Interpretation
Thoracic aorta	Wall thickening	Diffuse distal arch involvement	Worsening stenosis
Abdominal aorta	Below-renal narrowing	Fusiform dilation (11-20 mm)	New aneurysmal change
Subclavian arteries	Threadlike narrowing	Persistent luminal reduction	Stable
Carotid arteries	Left wall thickening	Bilateral involvement	New progression
Brachiocephalic artery	Normal	Wall thickening	Newly involved

Vascular surgery consultation in 2022 confirmed no contraindications to pregnancy, and surgical intervention was deemed unnecessary. The patient opted to delay conception due to chronic steroid use and known vascular risk, using timing-based behavioral contraception without hormonal agents. In 2025, she presented with left abdominal pain and vaginal bleeding. Transvaginal ultrasound confirmed a left tubal ectopic pregnancy with elevated β-hCG. She received intramuscular methotrexate and remained hemodynamically stable.

## Discussion

Takayasu arteritis (TA), often referred to as "pulseless disease," is a form of large vessel vasculitis. The clinical manifestations arise from ischemia caused by inflammation of major blood vessels, especially the aorta and its primary branches. Symptoms vary based on the specific vessels involved and may include headache, dizziness, fainting, weakened pulses, and differences in systolic blood pressure between the upper limbs [3]. However, atypical early manifestations and low incidence may lead to delayed diagnosis and serious, complex disease evolution, as this case illustrates.

The 2022 ACR/EULAR classification criteria were retrospectively applied, yielding diagnostic clarity in this atypical case [6]. The patient’s presentation included bilateral upper and lower extremity claudication, vascular bruits, diminished upper extremity pulses, a systolic blood pressure difference exceeding 20 mmHg between arms, and imaging-confirmed involvement of multiple aortic branches with paired vessel disease. This constellation of findings yielded a total score of 12 - well above the classification threshold (≥5) - thereby validating the initial clinical diagnosis.

Atypical otologic manifestations preceding vascular symptoms

This early atypical presentation highlights the patient’s initial symptom - isolated, persistent, bilateral pulsatile tinnitus. The tinnitus preceded systemic and vascular features by nearly five or six months. To further evaluate the auditory complaint, audiologic assessment included pure-tone audiometry, tympanometry, and MRI of the internal auditory canal and inner ear. Audiometry showed hearing thresholds within clinically accepted limits across all tested frequencies, with no air-bone gap. Tympanometry revealed normal middle ear pressure and compliance, and MRI showed no abnormalities.

Given the absence of structural or audiometric abnormalities, the underlying pathophysiology warrants further consideration. Potential mechanisms of auditory symptoms in Takayasu arteritis include autoimmune inflammation of the membranous labyrinth and vascular thrombosis of the labyrinthine artery and other small vessels [7]. Additional mechanisms seen in autoimmune diseases, such as middle ear effusions, Eustachian tube inflammation, ossicular chain dysfunction, and occlusion of labyrinthine or anterior vestibular arteries, may also contribute [8]. These insights reinforce the need to expand diagnostic considerations when evaluating unexplained otologic complaints, especially in young women.

Angiographic evolution and therapeutic milestones

Initial imaging conducted in Morocco in 2019 supported a type IIb angiographic classification. Despite ongoing corticosteroid therapy, follow-up MR angiography in 2022 performed in the United States revealed diffuse vascular changes, including fusiform dilation and new vessel involvement, aligning with type V, the most extensive pattern described in Takayasu arteritis (Table 4) [9]. This transition highlights the disease’s insidious progression and the critical need for longitudinal surveillance.

**Table 4:** Evolution of angiographic classification and vascular involvement from 2019 to 2022.

Year	Type	Vascular involvement
2019	Type IIb	Aortic arch, descending thoracic aorta, subclavian arteries, left carotid thickening
2022	Type V	Thoracoabdominal aorta, new aneurysmal segments, bilateral carotid, brachiocephalic involvement

The documented progression in angiographic findings not only illustrates the insidious nature of Takayasu arteritis but also satisfies key markers of disease activity. According to the National Institutes of Health (NIH) criteria, systemic symptoms, elevated inflammatory markers, and new arterial lesions are indicative of active disease, thereby justifying continuation of immunosuppressive therapy [10]. Subsequent CT angiography performed in 2024 revealed vascular stability, suggesting therapeutic control.

Ectopic gestation in Takayasu arteritis: an emerging concern

Pregnancy planning introduced another layer of complexity. Despite stable imaging, the patient's conception was delayed due to corticosteroid exposure and vascular risks. In 2025, she presented with a left-sided ectopic pregnancy and was medically managed with intramuscular methotrexate. No rupture occurred, and she remained hemodynamically stable. Following the event, reproductive counseling was reinforced with rheumatology and maternal-fetal medicine to reassess pregnancy planning and risk stratification. Ectopic pregnancy is an outcome seldom reported in TA. Most literature focuses on intrauterine complications such as miscarriage, preeclampsia, and fetal growth restriction [11]. The appearance of an ectopic gestation in this context highlights gaps in reproductive risk characterization for women with TA.

Immunovascular mechanisms underlying pregnancy risks

Vasoconstriction in Takayasu arteritis contributes to chronic uteroplacental insufficiency, exacerbation of existing hypertension, preeclampsia, and fetal growth restriction. Autoimmune inflammatory processes may impair placentation by targeting trophoblastic tissues, spiral artery endothelium, and decidual glandular cells, leading to disrupted vascular remodeling and reduced fetal perfusion [12]. While ectopic pregnancy is not widely characterized in TA, this case suggests a possible role for altered uterine or tubal perfusion and/or immunosuppressive therapy in abnormal implantation or tubal dysfunction. Expanded reproductive surveillance and tailored counseling are warranted.

This case exemplifies the need for coordinated care between rheumatology, vascular surgery, and maternal-fetal medicine. Future pregnancy planning should be guided by updated imaging, inflammatory activity markers, and shared decision-making. Longitudinal support can ensure safer outcomes and empower patients to navigate reproductive health within the context of systemic disease.

## Conclusions

We report a unique triad of isolated pulsatile tinnitus, progressive Takayasu arteritis, and ectopic pregnancy occurring under immunosuppressive therapy. This constellation broadens the recognized clinical spectrum of TA and underscores the importance of maintaining a high index of suspicion for vasculitis in patients presenting with unexplained auditory symptoms. Long-term disease management must be multidisciplinary, encompassing vascular, rheumatologic, and reproductive domains. Continuous clinical surveillance is essential to detect disease progression, treatment complications, and evolving reproductive risks. Further studies are warranted to understand the reproductive implications of TA better and to optimize care for young women affected by large-vessel vasculitis.
